# Multiple Sclerosis Patients Show Lower Bioavailable 25(OH)D and 1,25(OH)_2_D, but No Difference in Ratio of 25(OH)D/24,25(OH)_2_D and FGF23 Concentrations

**DOI:** 10.3390/nu11112774

**Published:** 2019-11-15

**Authors:** Mariska C Vlot, Laura Boekel, Jolijn Kragt, Joep Killestein, Barbara M. van Amerongen, Robert de Jonge, Martin den Heijer, Annemieke C. Heijboer

**Affiliations:** 1 Department of Clinical Chemistry, Endocrine Laboratory, Amsterdam University Medical Center, 1081 HV Amsterdam, The Netherlands; m.vlot@amsterdamumc.nl (M.C.V.); l.boekel@student.vu.nl (L.B.); r.dejonge1@amsterdamumc.nl (R.d.J.); 2 Department of Internal Medicine, Amsterdam UMC, Amsterdam University medical Center, 1081 HV Amsterdam, The Netherlands; m.denheijer@amsterdamumc.nl; 3 Department of Neurology, Reinier de Graaf Gasthuis, 2625 AD Delft, The Netherlands; J.Kragt@rdgg.nl; 4 Department of Neurology, Amsterdam Neuroscience, MS Center Amsterdam, Amsterdam University Medical Center, 1081 HV, Amsterdam, The Netherlands; j.killestein@amsterdamumc.nl; 5 Department of Molecular Cell Biology and Immunology, Amsterdam University Medical Center, Vrije Universiteit Amsterdam, 1081 HV Amsterdam, The Netherlands; bmvanamerongen@gmail.com; 6 Department of Clinical Chemistry, Endocrine Laboratory, Amsterdam University Medical Center, 1105 AZ Amsterdam, The Netherlands

**Keywords:** multiple sclerosis, fibroblast growth factor 23, vitamin D metabolites, vitamin D binding protein

## Abstract

Vitamin D (VitD) insufficiency is common in multiple sclerosis (MS). VitD has possible anti-inflammatory effects on the immune system. The ratio between VitD metabolites in MS patients and the severity of the disease are suggested to be related. However, the exact effect of the bone-derived hormone fibroblast-growth-factor-23 (FGF23) and VitD binding protein (VDBP) on this ratio is not fully elucidated yet. Therefore, the aim is to study differences in total, free, and bioavailable VD metabolites and FGF23 between MS patients and healthy controls (HCs). FGF23, vitD (25(OH)D), active vitD (1,25(OH)_2_D), inactive 24,25(OH)D, and VDBP were measured in 91 MS patients and 92 HCs. Bioavailable and free concentrations were calculated. No difference in FGF23 (*p* = 0.65) and 25(OH)D/24.25(OH)_2_D ratio (*p* = 0.21) between MS patients and HCs was observed. Bioavailable 25(OH)D and bioavailable 1.25(OH)_2_D were lower (*p* < 0.01), while VDBP concentrations were higher in MS patients (*p* = 0.02) compared with HCs, specifically in male MS patients (*p* = 0.01). In conclusion, FGF23 and 25(OH)D/24.25(OH)_2_D did not differ between MS patients and HCs, yet bioavailable VitD concentrations are of potential clinical relevance in MS patients. The possible immunomodulating role of VDBP and gender-related differences in the VD-FGF23 axis in MS need further study.

## 1. Introduction

Multiple sclerosis (MS) is a chronic, progressive disease of the central nervous system characterized by an inflammatory, demyelinating, and neurodegenerative process, which can result in varying levels of disability. Consequently, MS has a negative effect on the daily life of patients, resulting from fatigue, muscle weakness, and imbalance to immobility, which all negatively affect bone health [1,2,3,4]. Earlier studies showed that decreased bone mineral density (BMD) is prevalent already shortly after clinical onset even in physically active patients with MS [2,4,5,6]. Lower BMD combined with impaired mobility can result in an increased fracture risk in MS patients and accompanying disability and economic burden [7]. The specific aetiology of MS is still unknown, but strong evidence exists regarding viral, genetic, and immunological causes of the disease [8,9].

Vitamin D is thought to play a role in the pathogenesis of MS, as it is known that the prevalence of MS increases with latitude, which in turn is associated with lower serum concentrations of vitamin D (25(OH)D) [10,11]. Vitamin D possibly modulates T-lymphocyte subset differentiation and, therefore, a lower concentration is thought to lead to an increased risk of MS [12]. In line with this, associations between lower serum concentrations 25(OH)D and an increased risk of MS are shown in several studies [13,14,15]. As a result, vitamin D supplementation is often advised to MS patients, although contra-dictionary effects regarding the beneficial effects of increased 25(OH)D concentrations on, for example, the recurrence rate of relapses, deterioration of number of brain laesions, and improvement of disability are described [16,17,18,19,20]. Because of the potential role of vitamin D in MS, other vitamin D metabolites have been studied in MS patients. 25(OH)D needs to be metabolized to the biologically active 1.25(OH)_2_D, and both metabolites are predominantly bound to their carrier vitamin D binding protein (VDBP). Previous research showed a higher plasma VDBP concentration in MS patients compared with healthy controls (HCs) [21]. VDBP is known to play an important role in the intracellular actin scavenging system by removing actin derived from damaged tissue and also promotes inflammation [22,23,24,25,26]. Polymorphisms of both VDBP and also of the vitamin D receptor (VDR) can result in a changed equilibrium between active and inactive vitamin D metabolites [24,27,28]. Interestingly, lower concentrations of the vitamin D metabolite 24.25(OH)_2_D were associated with a higher grade of disability based on the Expanded Disability Status Scale (EDSS) score in MS patients [29]. Moreover, the ratio of 25(OH)D/24.25(OH)_2_D was strongly inversely associated with brain parenchymal function [29].

A key player in vitamin D metabolism is the bone-derived hormone fibroblast growth factor 23 (FGF23), as it inhibits the enzyme 1-alpha hydroxylase and stimulates the enzyme 24-hydroxylase, resulting in the conversion of 25(OH)D into 24.25(OH)_2_D instead of into 1.25(OH)_2_D. We thus hypothesize that plasma FGF23 concentrations differ between MS patients and healthy controls.

The primary aim of this study is to further elucidate the vitamin D-FGF23 axis by measuring multiple D metabolites and FGF23 using accurate state-of-the art analytical methods in a well-defined cohort of MS patients and healthy controls. The second aim of our study is to assess bone turnover markers (BTMs) in our cohort MS patients and healthy controls and to study the possible associations of BTMs with vitamin D metabolites, as vitamin D deficiency is associated with increased bone turnover and lower bone mineral density [30,31,32]. Lastly, it is known that MS affects more women than men and that women have higher VDBP concentrations compared with men [13,21,33,34,35,36]. Therefore, possible gender-related differences of vitamin D metabolites and BTMs between MS patients and healthy controls will be studied.

## 2. Materials and Methods

### 2.1. Subjects and Study Protocol

The subjects and study protocol were described earlier by Kragt et al. [13]. Patients were eligible to participate in the study if they provided informed consent and were between 18 and 75 years of age. Three subtypes of MS were identified: relapsing-remitting MS (RRMS), secondary progressive MS (SPMS), and primary progressive MS (PPMS). Patients with all subtypes of MS were eligible to participate. In addition, patients with clinically isolated syndrome (CIS) were also included, which refers to a single episode of symptoms that are suggestive for MS. Patients were recruited between July and September 2003, to ensure that all blood samples were drawn during summer season. Patients were asked to bring a healthy control with them to the outpatient clinic if possible, preferably their partner in order to match the patients based on age and environmental factors. If a healthy control was lacking, hospital personnel volunteered to participate as controls. Patients were excluded if they were diagnosed with osteomalacia, hyperparathyroidism, hyperthyroidism, or hypercortisolism, or if they had been receiving glucocorticoid treatment in the previous three weeks (daily oral treatment or intravenous methylprednisolone treatment), anti-epileptic drugs, or vitamin D supplementation of more than 200 IU/day. The controls were excluded if they had MS or had a first-degree family member with MS. In addition, study participants were excluded in the case of suspicion of concomitant bone disease based on their laboratory results. All subjects gave their informed consent for inclusion before they participated in the study. The study was conducted in accordance with the Declaration of Helsinki, and the protocol was approved by the Medical Ethical Committee of the Amsterdam UMC, location VU University Medical Center (code 2003.029).

### 2.2. Measurements

#### 2.2.1. General

The functional disability of MS patients was evaluated using the Expanded Disability Status Scale (EDSS) [37]. EDSS quantifies disability in MS patients, which ranges from 0 to 10, with higher scores representing higher levels of disability. Blood samples were collected before 00:00 and after an overnight fast. Samples were collected in 2003 and stored (both as serum and EDTA plasma in several aliquots) in minus 80 degrees Celsius. Analyses were performed in 2011 (parathyroid hormone (PTH), alkaline phosphatase (ALP), albumin, calcium, phosphate, estimated glomerular filtration rate (eGFR)) and in 2017 (25(OH)D, 24.25(OH)_2_D, 1.25(OH)_2_D, VDBP, FGF23, c-terminal telopeptide (CTX), osteocalcin, procollagen type 1 N-terminal propeptide (P1NP)) in the various aliquots.

#### 2.2.2. Vitamin D Metabolites

In the current study, 25(OH)D and 24.25(OH)_2_D were measured in serum using a dedicated isotope dilution liquid chromatography-tandem mass spectrometry (ID-LC-MS/MS) [38]. Total serum concentrations of 25(OH)D were calculated by the sum of 25(OH)D_2_ and 25(OH)D_3_. For 25(OH)D_2_, the lower limit of quantitation (LLOQ) was 0.36 nmol/L and the inter-assay coefficient of variation (CV) was 6%. For 25(OH)D_3_, LLOQ was 1.19 nmol/L and the inter-assay CV was 6%. For 24.25(OH)_2_D, LLOQ was 0.12 nmol/L and the inter-assay CV was 5% [37,39]. A two-dimensional isotope dilution ultra-pressure liquid chromatography tandem mass spectrometry (2D ID-UPLC-MS/MS) was used to measure serum 1.25(OH)_2_D with an LLOQ of 3.4 pmol/L and inter-assay CV of 11% [40]. It is of note that, in the earlier study of Kragt et al., a radioimmunoassay was used for the measurement of 25(OH)D and 1.25(OH)_2_D [13], but the concentrations shown in the current study are obtained using an LC-MS/MS method. VDBP was measured using a polyclonal ELISA (Immundiagnostik AG) with a LLOQ of 2.2 µg/L and an inter-assay CV of <13%.

#### 2.2.3. Free and Bioavailable Vitamin D Metabolites

Vitamin D metabolites bound to protein are not biologically active, whereas the unbound hormones are. Free 25(OH)D was calculated using equations and affinity constants according to Malmstroem et al. [41] and free 1.25(OH)_2_D was calculated using equations and affinity constants according to Bikle et al. [42]. Bioavailable 25(OH)D and 1.25(OH)_2_D were calculated using equations adapted from Vermeulen et al. [43] and the supplement of Powe et al. [44].

#### 2.2.4. Bone Turnover Markers (BTMs)

FGF23 was measured in EDTA plasma using a c-terminal immunoassay (Immutopics) with an LLOQ of 20 RU/mL and inter-assay CV of <10% [45]. CTX and P1NP were measured in EDTA plasma using an immunoassay (Cobas, Roche Diagnostics, Almere, The Netherlands), with an LLOQ of 10 ng/L and inter-assay CV of <6.5% for CTX and an LLOQ of 5 µg/L and inter-assay CV of <8% for P1NP, respectively. Osteocalcin was measured in EDTA plasma using an immunometric-assay (Biosource, Nivelles, Belgium) with an LLOQ of 0.4 nmol/L and inter-assay CV of 8–15%.

#### 2.2.5. Other Measurements

PTH was measured in EDTA plasma using an immunoassay (Architect, Abbott Diagnosher, Chicago, IL, USA) with an LLOQ 0.5 pmol/L and inter-assay CV of <9%. ALP, eGFR, calcium, phosphate, and albumin were measured in heparin plasma using the chemistry module of the Cobas (Roche Diagnostics).

### 2.3. Statistical Analysis

Baseline characteristics between MS patients and healthy controls were compared using a Student’s t-test or chi-square test. The Mann–Whitney U test was used in the case of non-parametric variables. To detect differences between patients and controls in biochemical indices of the vitamin D metabolites and BTMs, the Mann–Whitney U test was used as well. In addition, separate analyses for men and women were performed. Lastly, correlations between serum vitamin D metabolites, BTMs, and EDSS were calculated using Spearman correlations owing to non-parametric variables. All statistical analyses were carried out using SPSS software, version 23.0.

## 3. Results

### 3.1. General

**Figure 1:**
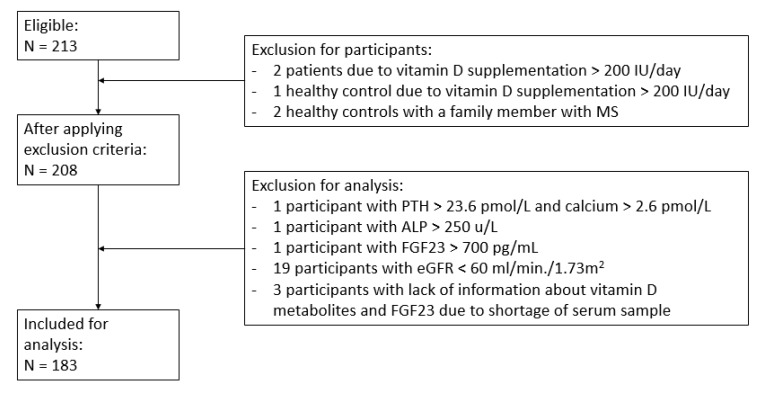
Flow chart of study population selection. PTH = parathyroid hormone, ALP = alkaline phosphatase, eGFR = estimated glomerular filtration rate FGF23 = fibroblast growth factor 23, MS = multiple sclerosis.

A total of 213 participants were eligible to be included in this study. After applying the in- and exclusion criteria, a total of 30 participants were excluded; see Figure 1. The final study population that was included in the analyses consisted of 183 participants based on 91 MS patients and 92 healthy controls. Table 1 summarizes the baseline characteristics of all participants. In total, 61% of the MS patients were female. Relapsing remitting MS was the most predominant subtype of MS (57%).

### 3.2. Vitamin D Metabolites, FGF23, and Bone Turnover Markers

**Figure 2:**
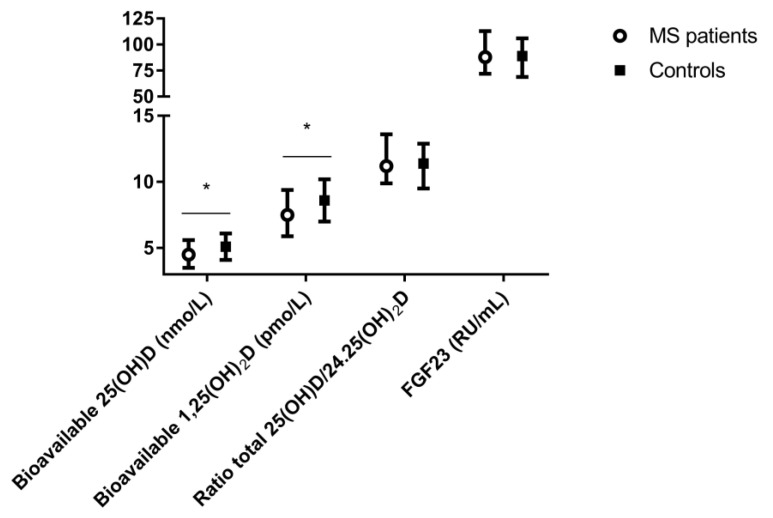
Vitamin D metabolites and FGF23 in MS patients versus healthy controls, * *p* ≤ 0.05.

Biochemical indices of vitamin D metabolism and bone turnover of the study population are displayed in Table 1. Again, it is of note that, in contrast to the earlier study of Kragt et al., current 25(OH)D and 1.25(OH)_2_D measurements are reported based on the ID-LC-MS/MS measurements [13]. Overall, no significant difference between total serum concentrations of 25(OH)D (*p* = 0.06) was found between MS patients and the healthy controls. MS patients had significant lower serum concentrations of free, albumin bound, and bioavailable 25(OH)D and 1.25(OH)_2_D compared with healthy controls (*p* < 0.01). In addition, MS patients had higher concentrations of phosphate and VDBP compared with controls, whereas no significant differences in BTMs were found. Plasma FGF23 concentrations did not differ between MS patient and the healthy controls (*p* = 0.65) (Figure 2).

Table 2 shows the significant results of separate analyses for men and women. In contrast to male patients, female MS patients had lower serum concentrations of total 25(OH)D, 25(OH)D_3_, 24.25(OH)_2_D, free 25(OH)D, and free 1.25(OH)_2_D compared with healthy female controls (*p* = 0.04). Male MS patients had higher serum concentrations of VDBP compared with male controls (*p* = 0.01). No other significant differences were found between male MS patients and healthy male controls. Regarding BTMs, no significant differences were found between males and females (data not shown). Lastly, EDSS scores did not differ between male and female MS patients (*p* = 0.77, data not shown).

### 3.3. Associations

#### 3.3.1. Associations between Vitamin D Metabolites, FGF23, Bone Turnover Markers, and EDSS in MS Patients

In MS patients, all vitamin D metabolites correlated strongly with each other (all *r* > 0.77; *p* < 0.01, data not shown). EDSS showed negative correlations with bioavailable 1.25(OH)_2_D, bioavailable 25(OH)D, and 24.25(OH)_2_D (*r* −0.30, *p* < 0.01; *r* −0.30, *p* < 0.01; and *r* −0.23, *p* = 0.03, respectively). No correlations between EDSS and FGF23 or BTMs in MS patients were found (data not shown).

As shown in Table 3, FGF23 correlated positively with serum 25(OH)D and serum 24.25(OH)_2_D (*r* 0.22, *p* = 0.04 and *r* 0.22, *p* = 0.04, respectively), and CTX correlated negatively with serum 24.25(OH)_2_D and 25(OH)D (*r* −0.31, *p* < 0.01 and *r* −0.23, *p* = 0.03, respectively) in MS patients. Phosphate showed a positive correlation with osteocalcin (*r* 0.22, *p* 0.04). Negative correlations for osteocalcin with 1.25(OH)_2_D, and P1NP with 24.25(OH)_2_D, were observed in MS patients only (*r* −0.24, *p* = 0.02 and *r* −0.25, *p* = 0.02, respectively), as were positive correlations of the ratio between total serum 25(OH)D and 24.25(OH)_2_D with P1NP and CTX (*r* 0.27, *p* = 0.01 and *r* 0.31, *p* < 0.01, respectively). In addition, in MS patients, ALP showed a positive correlation with osteocalcin, CTX, and P1NP (*r* 0.36, *p* < 0.01; *r* 0.44, *p* < 0.01; and *r* 0.43, *p* < 0.01, respectively); P1NP showed a positive correlation with osteocalcin and CTX (*r* 0.75, *p* < 0.01 and *r* 0.74, *p* < 0.01, respectively); and CTX correlated positively with osteocalcin (*r* 0.67, *p* < 0.01).

#### 3.3.2. Associations Based on Gender of MS Patients

Looking at gender differences, in female MS patients, the same correlations were found as described above, except for the correlations of FGF23 with serum 25(OH)D and 24.25(OH)_2_D (*r* 0.12, *p* = 0.37 and *r* 0.10, *p* = 0.44, respectively). In male MS patients, however, FGF23 correlated positively with total (*r* 0.50, *p* < 0.01), free (*r* 0.43, *p* = 0.02), and bioavailable 25(OH)D (*r* 0.43, *p* = 0.02); free (*r* 0.43, *p* 0.02) and bioavailable 1.25(OH)_2_D (*r* 0.43, *p* 0.02); and 24.25(OH)_2_D (*r* 0.46, *p* = 0.01), respectively. Furthermore, EDSS showed negative correlations with CTX (*r* −0.53, *p* < 0.01), P1NP (*r* −0.48, *p* < 0.01), and osteocalcin (*r* −0.40, *p* 0.03) in male MS patients only.

#### 3.3.3. Associations in Healthy Controls

Lastly, in healthy controls, similar correlations of osteocalcin, CTX, and P1NP were found (data not shown). FGF23 correlated significantly with serum 1.25(OH)_2_D (*r* −0.35, *p* < 0.01; data not shown), and CTX correlated significantly with serum 25(OH)D (*r* −0.24, *p* = 0.05; data not shown). In addition, PTH showed a positive correlation with ALP (r 0.21, p 0.04; data not shown) and phosphate showed a negative correlation with P1NP (*r* −0.27, *p* 0.03; data not shown) in the healthy control group.

## 4. Discussion

This study examined differences in total, free, and bioavailable vitamin D, FGF23 and bone turnover markers in patients with MS compared with healthy controls and possible gender differences. Although positive correlations between FGF23 and total 25(OH)D and 24.25(OH)_2_D in MS patients were seen, no differences in plasma FGF23 concentrations between MS patients and healthy controls were observed. No differences between serum concentrations of BTMs in MS patients and healthy controls were found. Yet, we did observe a negative correlation between CTX and total 25(OH)D and 24.25(OH)_2_D. EDSS showed negative correlations with bioavailable 1.25(OH)2D, bioavailable 25(OH)D, and 24.25(OH)2D. We found gender differences in vitamin D metabolism: serum concentrations of total 25(OH)D, 25(OH)D_3_, 24.25(OH)_2_D, free 25(OH)D, and free 1.25(OH)_2_D were lower in female MS patients compared with female healthy controls. Serum concentrations of VDBP were higher in male MS patients compared with healthy male controls.

The main aim of this study was to further elucidate the vitamin D-FGF23 axis by measuring multiple D metabolites and FGF23 using accurate state-of-the art analytical methods in a well-defined cohort of MS patients versus healthy controls. We confirmed the finding of an earlier study that the serum 24.25(OH)_2_D concentration is negatively correlated with EDSS [29]. Although we found a significant positive correlation between FGF23 and 25(OH)D and 24.25(OH)_2_D, we did not observe a difference in FGF23 concentration between MS patients and healthy controls. A previous study described comparable plasma concentrations of FGF23 in MS patients compared with healthy controls as well [46], although two other studies found higher serum concentrations of FGF23 in MS patients compared with healthy controls [47,48]. However, the latter study was performed in patients with RRMS only and these differences were found during autumn (September–November) and winter time, respectively. Therefore, seasonal effects or the use of different FGF23 assays measuring either intact or c-term FGF23 could have resulted in these different findings. We found a higher phosphate concentration in MS patients; nevertheless, no differences in PTH or eGFR compared with healthy controls were found. Lastly, no correlations between FGF23 and 1.25(OH)_2_D or EDSS were found in MS patients, which is in line with two other recent studies [46,48]. Summarized, these findings do not support our hypothesis that FGF23 differs between MS patients and healthy controls.

Regarding the various vitamin D metabolites, we found similar concentrations of 25(OH)D and 1.25(OH)_2_D between MS patients and healthy controls, as some showed earlier [13,49,50], whereas others found lower serum concentrations of 25(OH)D or 1.25(OH)_2_D in MS patients compared with healthy controls, respectively [15,51,52,53,54,55,56,57]. These differences between studies might be caused by differences in sample size, analysis of both male and female participants in different seasons of the year, use of different assays to measure vitamin D and its metabolites, and possible VDBP polymorphisms [24,27,28]. In addition, a number of studies did report a relationship between lower serum concentrations of 1,25(OH)_2_D and an increased risk of MS [51,52]. Furthermore, our study showed that MS patients were using supplementation of vitamin D more often compared with their healthy controls. However, the maximal supplementation dosage was <200 IU of vitamin D per day, of which no clinical relevant effect on the vitamin D metabolites is expected, as a higher supplementation dosage is normally advised [18,19,29]. Moreover, controls had similar baseline concentrations of 25(OH)D.

As suggested in recent studies, bioavailable 25(OH)D might be preferred above total 25(OH)D as a marker for vitamin D status, and might thus be a better marker of mineral metabolism [58,59,60,61,62]. The sum of the albumin bound fraction of 25(OH)D plus the freely circulating 25(OH)D results in the bioavailable 25(OH)D, which can be calculated similarly for bio-available 1.25(OH)_2_D, respectively [58,60]. In the circulation, VDBP and albumin bind over 99% of the 25(OH)D and 1.25(OH)_2_D [60], whereas the binding affinity of vitamin D to albumin is much lower than the binding affinity to VDBP [63]. We found lower serum concentrations of both free and bioavailable 25(OH)D and 1.25(OH)_2_D, respectively, in MS patients compared with healthy controls, in contrast to comparable serum concentrations of total 25(OH)D and 1.25(OH)_2_D between MS patients and healthy controls. In contrast to our findings, Behrens et al. found no difference in free and bioavailable 25(OH)D between MS patients and controls, yet 1.25(OH)_2_D was not measured in this study [64]. However, their study included patients with CIS only (not yet diagnosed with MS), used non-parametrical statistical tests, and a de-seasonalized concentration of vitamin D was calculated afterwards. Our study measured summer concentrations of vitamin D only and included all subtypes of MS, which may explain the different findings.

The second aim of our study was to assess BTMs in MS patients versus healthy controls and to study the possible associations of BTMs with vitamin D metabolites, as vitamin D deficiency is associated with increased bone turnover [32]. Our study did not show any differences between BTMs and FGF23 in MS patients and their healthy controls. This finding is in line with earlier studies, where similar serum concentrations of CTX, P1NP, or osteocalcin between MS patients and healthy controls were found, and similar concentrations of cross-linked N terminal telopeptide type 1 collagen (NTX) and bone ALP in newly diagnosed MS patients and healthy controls were found [18,32,65,66,67]. The current study shows that, despite differences in vitamin D metabolites, bone turnover in MS patients seems to not be affected compared with healthy controls.

Lastly, possible gender-related differences of vitamin D metabolites and BTMs between MS patients and healthy controls were studied; as known, MS affects more women than men and, in general, women have higher VDBP concentrations compared with men [13,21,33,34,35,36]. Indeed, our study showed more female MS patients, of which the largest part was post-menopausal. Further analyses based on pre- or postmenopausal status was not possible because of small groups. Our study showed lower total and free 25(OH)D, free 1.25(OH)_2_D, and 24.25(OH)_2_D only in female MS patients compared with healthy female controls, but no difference in this respect between male MS patients compared with healthy men. No differences between FGF23 in male and female MS patients were found. Interestingly, in our study, VDBP was higher in MS patients compared with controls, but after further analysis, this difference was found in male MS patients only. An earlier study found that serum VDBP concentrations were higher in MS patients compared with healthy controls as well; however, this study included both female and males [21]. In contrast, other studies showed no difference in VDBP concentrations between MS patients and controls [64,68]. The differences between VDBP concentrations in the various studies could be the result of variation between numbers of male and female patients and controls included in these studies or by using either a polyclonal or monoclonal assay. It is known that VDBP can affect inflammatory processes [22,23,25,26,61,69,70]. As we found higher VDBP in male MS patients, in the presence of a similar EDSS score as in female MS patients, this suggests a possible modulating effect of VDBP in male MS patients. It was shown before that higher concentrations of VDBP restrict the uptake of free vitamin D metabolites and reduce anti-inflammatory responses of immune cells [71,72,73,74]. Moreover, VDBP can act as a chemotactic cofactor, which enhances chemotaxis of neutrophils and macrophages by complement factor C5a [23]. These macrophages are known to contribute to laesion formation and axonal damage [22,26]. Previous experimental studies showed that T-lymphocytes, glia cells, and neurons express 1-α-hydroxlyase and VDR, which enables them to convert 25(OH)D to 1.25(OH) [75,76,77]. Normally, in the central nervous system, focal inflammation is initiated by auto-reactive T-helper cells type 1 (Th 1) and T-helper cells type 17 (Th 17) [78]. The activated vitamin D is thought to induce anti-inflammatory effects in glia cells and neurons and affects vitamin D responsive genes in T-helper cells [76,79,80,81,82], thereby inhibiting pro-inflammatory T-helper cell activity (Th1 and Th 17) and promoting anti-inflammatory T-helper cell activity (Th2 and regulatory T-cells) [51,83]. However, whether vitamin D affects the T-cell response also depends on concentrations of VDBP [84,85]. On the basis of these studies, the elevated concentrations of VDBP found in our study strengthen the pro-inflammatory role of VDBP in enhancing neuronal damage in MS.

Lastly, EDSS scores were comparable between male and female MS patients, which is consistent with previous research [86,87]. In female MS patients, however, EDSS showed negative correlations with free and bioavailable 1.25(OH)_2_D; free, bioavailable, and total 25(OH)D; and 24.25(OH)_2_D, which is in line with earlier studies, which reported a negative correlation between EDSS and 1.25(OH)_2_D or 25(OH)D, respectively [46,88]. In contrast, in male MS patients, negative correlations between EDSS and CTX, P1NP, and osteocalcin were observed, which differs from previous studies, which found no correlations between EDSS and BTMs or only a positive correlation between EDSS and CTX [67,89].

The strengths of this study are the relative large study population and the broad spectrum of vitamin D metabolites, FGF23, and BTMs that was measured. Accurate and well-standardized LC-MS/MS assays were used to measure vitamin D metabolites. In addition, to minimize seasonal changes known to affect vitamin D metabolism [48], only blood samples drawn during summertime were used. There are also some possible limitations of this study. First, owing to the cross-sectional study design, the described differences between MS patients and controls in serum concentrations of vitamin D metabolites are not necessarily causal. In addition, 97% of our study population is Caucasian, which reduces the generalizability of the results, as it is known that vitamin D metabolism differs between races [53]. Moreover, our study was not primarily designed to study gender difference, so the differences we found in this respect should be studied further in other cohorts. No data on fractures were available. Lastly, dual energy X-ray absorptiometry (DXA) scans were not available to compare vitamin D metabolites and bone turnover markers with BMD.

## 5. Conclusions

In conclusion, this study provides additional knowledge of vitamin D-FGF 23 axis and bone turnover markers in MS patients compared with healthy controls, as well as gender-related differences. Similar serum concentrations of total 25(OH)D and 1.25(OH)_2_D in MS patients and healthy controls were found. Furthermore, no differences in plasma FGF23 concentrations and other bone turnover markers between MS patients and healthy controls were observed. The ratio total 25(OH)D/ 24.25(OH)_2_D did not differ between MS patients and healthy controls. However, this study suggested a relevant gender difference as serum concentrations of total 25(OH)D, 24.25(OH)_2_D, free 25(OH)D, and free 1.25(OH)_2_D were lower in female MS patients compared with female healthy controls, while serum concentrations of VDBP were higher in male MS patients compared with male controls. This study thus shows that only a total 25(OH)D measurement probably does not reflect all changes in vitamin D metabolism in MS patients. The exact role of VDBP and its polymorphisms in MS needs further studies. Finally, given the high incidence of reduced bone mineral density and the still partially unknown mechanisms that affect bone turnover in MS patients [66], further studies should be performed to evaluate the relationship between change in the vitamin D-FGF23 axis, BMD, and fracture risk in both male and female MS patients.

## Figures and Tables

**Table 1:** Baseline characteristics, vitamin D metabolites, FGF23, and bone turnover markers of multiple sclerosis (MS) patients and controls displayed as median with corresponding interquartile range (IQR), unless specified otherwise, of MS patients and controls.

	Patients (*n* = 91)	Controls (*n* = 92)	*p* Value ^a^	*Reference Range*
Age, yr (mean ± SD)	45 ± 11	42 ± 11	0.30	
Female of total population (%)	67	41	<0.01 *	
Postmenopausal of total population (%)	34	16	0.04 *	
Caucasian (%)	98	95	0.44	
eGFR, mL/min/1.73m^2^ (median, IQR)	71 (66–81)	71 (67–78)	0.42	>60
MS subtype (%)		n.a.		
RRMS	57			
SPMS	24			
PPMS	14			
CIS	1			
Disease duration, yr (median, IQR)	10 (5–16)	n.a.		
EDSS (median, IQR)	4 (3–6)	n.a.		
Use of vitamin D supplements ^b^, (%)	40	14	<0.01 *	
Use of using disease modifying therapy, (%)	32	n.a.		
Total 25(OH)D, nmol/L	75 (59–93)	77 (67–98)	0.06 ^#^	>50
Total 1.25(OH)_2_D, pmol/L	105 (74–143)	99 (79–133)	0.57	59–159
25(OH)D_2_, nmol/L	1.1 (0.8–1.5)	1.2 (0.9–1.5)	0.66	
25(OH)D_3_, nmol/L	73 (58–92)	76 (66–96)	0.05 ^#^	
24.25(OH)D, nmol/L	6.5 (4.4–9.0)	7.1 (5.4–9.6)	0.08 ^#^	0.4–8.9
Free 25(OH)D (* 10^−2^), nmol/L	1.2 (1.0–1.5)	1.4 (1.2–1.7)	<0.01 *	
Albumin bound 25(OH)D, nmol/L	4.5 (3.5–5.6)	5.1 (4.1–6.1)	<0.01 *	
Bioavailable 25(OH)D, nmol/L	4.5 (3.5–5.6)	5.1 (4.1–6.1)	<0.01 *	
Free 1.25(OH)_2_D (* 10^−1^), pmol/L	2.0 (2.2–2.8)	2.5 (0.2–3.1)	<0.01 * ^#^	
Albumin bound 1.25(OH)_2_D, pmol/L	7.3 (5.7–9.1)	8.4 (6.7–9.9)	<0.01 *	
Bioavailable 1.25(OH)_2_D, pmol/L	7.5 (5.9–9.4)	8.6 (7.0–10.2)	<0.01 *	
Ratio total 25(OH)D/24.25(OH)_2_D	11.2 (9.9–13.6)	11.4 (9.5–12.9)	0.21	10–33
VDBP, µg/L	408 (374–445)	388 (361–427)	0.02 * ^#^	200–550
Albumin, g/L	42 (39–44)	42 (40–43)	0.86	35–52
Calcium, mmol/L	2.4 (2.3–2.4)	2.4 (2.3–2.4)	0.73	2.2–2.6
Corrected calcium, mmol/L	2.3 (2.3–2.4)	2.3 (2.3–2.4)	0.81	
FGF23, RU/mL	88 (72–113)	89 (69–106)	0.65	<125
PTH, pmol/L	5.2 (4.0–6.6)	5.3 (3.8–6.7)	0.94	<10
Phosphate, mmol/L	1.0 (0.9–1.1)	0.8 (0.8–0.9)	<0.01 *	0.7–1.4
CTX, ng/L	256 (183–379)	307 (212–418)	0.10	<580
P1NP, µg/L	37 (27–54)	39 (29–56)	0.37	22–87
ALP, U/L	74 (53–89)	67 (56–79)	0.14	<115
Osteocalcin, nmol/L	1.5 (1.1–2.1)	1.5 (1.1–2.0)	0.99	0.4–4.0

eGFR = estimated glomerular filtration rate, RRMS = relapsing remitting MS, SPMS = secondary progressive MS, PPMS = primary progressive MS, CIS = clinically isolated syndrome, SP + R = secondary progressive + remitting, EDSS = Expanded Disability Status Scale, VDBP = vitamin D binding protein, PTH = parathyroid hormone, ALP = alkaline phosphatase, FGF23 = fibroblast growth factor 23, CTX = c-terminal telopeptide, P1NP = procollagen type 1 N-terminal propeptide. ^a^ *p* values of Student’s t-test, chi-square test, or Mann–Whitney U test. ^b^ Maximum allowed dose of vitamin D supplements was 200 IU/day. * Bold, significance level *p* ≤ 0.05. ^#^ Significant difference between male and female; details can be found in Table 2.

**Table 2:** Vitamin D metabolites of MS patients and controls stratified for gender, only shown in the case of significant differences between male and females, displayed as median with corresponding interquartile range (IQR).

	Men (*n* = 84)			Women (*n* = 99)		
	Patients (*n* = 30)	Controls (*n* = 54)	*p* values ^a^	Patients (*n* = 61)	Controls (*n* = 38)	*p* values ^a^
Total 25(OH)D, nmol/L	74 (56–97)	75 (66–90)	0.62	77 (60–90)	88 (68–106)	0.02 *
25(OH)D_3_, nmol/L	73 (55–96)	74 (64–89)	0.64	75 (58–89)	86 (67–104)	0.01 *
24.25(OH)_2_D, nmol/L	6.5 (4.5–8.8)	6.9 (5.4–8.7)	0.48	6.5 (4.4–9.1)	7.1 (5.6–11.0)	0.04 *
Free 25(OH)D (* 10^−2^), nmol/L	0.013 (0.010–0.017)	0.014 (0.012–0.017)	0.17	0.012 (0.010–0.015)	0.013 (0.011–0.017)	0.03 *
Free 1.25(OH)_2_D (* 10^−1^), pmol/L	0.23 (0.17–0.30)	0.25 (0.22–0.31)	0.18	0.21 (0.17–0.27)	0.24 (0.21–0.31)	0.03 *
Phosphate, mmol/L	1.0 (0.9–1.0)	0.8 (0.8–0.9)	<0.01 *	1.0 (0.9–1.1)	0.8 (0.8–0.9)	<0.01 *

VDBP = vitamin D binding protein, PTH = parathyroid hormone. ^a^ *p* values of Mann–Whitney U test are shown as medians with interquartile ranges in parentheses. * bold significance level *p* ≤ 0.05.

**Table 3:** Correlation between vitamin D metabolites, bone turnover markers (BTMs), and EDSS in MS patients.

*N* = 90	ALP	FGF23	Osteocalcin	CTX	P1NP	EDSS
Free 1.25(OH)_2_D	*r* −0.03	*r* 0.10	*r* 0.01	*r* −0.10	*r* −0.07	***r* −0.28**
	*p* 0.78	*p* 0.33	*p* 0.95	*p* 0.33	*p* 0.54	***p* 0.01**
Bioavailable 1.25(OH)_2_D	*r* −0.04	*r* 0.10	*r* 0.04	*r* −0.06	*r* −0.03	***r* −0.30**
	*p* 0.71	*p* 0.33	*p* 0.72	*p* 0.59	*p* 0.77	***p* < 0.01**
Total 1.25(OH)_2_D	*r* −0.12	*r* −0.05	***r* −0.24**	*r* −0.09	*r* −0.19	*r* −0.08
	*p* 0.25	*p* 0.65	***p* 0.02**	*p* 0.41	*p* 0.08	*p* 0.47
Free 25(OH)D	*r* −0.02	*r* 0.10	*r* 0.01	*r* −0.10	*r* −0.06	***r* −0.28**
	*p* 0.82	*p* 0.34	*p* 0.93	*p* 0.36	*p* 0.57	***p* 0.01**
Bioavailable 25(OH)D	*r* −0.03	*r* 0.11	*r* 0.05	*r* −0.05	*r* −0.02	***r* −0.30**
	*p* 0.77	*p* 0.33	*p* 0.66	*p* 0.67	*p* 0.84	***p* < 0.01**
Total 25(OH)D	*r* −0.16	***r* 0.22**	*r* −0.12	***r* −0.23**	*r* −0.19	***r* −0.23**
	*p* 0.14	***p* 0.04**	*p* 0.28	***p* 0.03**	*p* 0.08	***p* 0.03**
24.25(OH)D	*r* −0.20	***r* 0.22**	*r* −0.16	***r* −0.31**	***r* −0.25**	***r* −0.22**
	*p* 0.06	***p* 0.04**	*p* 0.14	***p* < 0.01**	***p* 0.02**	***p* 0.04**
Ratio 25(OH)D/24.25(OH)_2_D	*r* 0.18	*r* −0.13	*r* 0.16	***r* 0.31**	***r* 0.27**	*r* 0.10
	*p* 0.09	*p* 0.23	*p* 0.13	***p* <0.01**	***p* 0.01**	*p* 0.34
PTH	*r* 0.09	*r* −0.04	*r* −0.04	*r* 0.04	*r −0.08*	*r* 0.16
	*p* 0.40	*p* 0.69	*p* 0.97	*p* 0.69	*p* 0.46	*p* 0.13
Phosphate	*r* 0.10	*r* 0.03	***r* 0.22**	*r* 0.13	*r* 0.16	*r* −0.03
	*p* 0.35	*p* 0.78	***p* 0.04**	*p* 0.24	*p* 0.15	*p* 0.76

ALP = alkaline phosphatase, FGF23 = fibroblast growth factor 23, CTX = c-terminal telopeptide, P1NP = procollagen type 1 N-terminal propeptide, EDSS = Expanded Disability Status Scale, PTH = parathyroid hormone. Bold text indicates a significance level of *p* ≤ 0.05. *r*: Spearman correlation coefficient.
